# Adaptive Event-Triggered Consensus of Multi-Agent Systems in Sense of Asymptotic Convergence

**DOI:** 10.3390/s24020339

**Published:** 2024-01-06

**Authors:** Zhicheng Hou, Zhikang Zhou, Hai Yuan, Weijun Wang, Jian Wang, Zheng Xu

**Affiliations:** 1 School of Automation, Guangdong Polytechnic Normal University, Guangzhou 523952, China; zhicheng.hou@gpnu.edu.cn (Z.H.); zhikang.zhou@hotmail.com (Z.Z.); 2 Guangzhou Institute of Advanced Technology, Guangzhou 511458, China; hai.yuan@giat.ac.cn (H.Y.); jian.wang@giat.ac.cn (J.W.); zheng.xu@giat.ac.cn (Z.X.); 3 University of Chinese Academy of Sciences, Beijing 100049, China

**Keywords:** multi-agent systems, event-triggered control, adaptive strategy, asymptotic consensus

## Abstract

In this paper, the asymptotic consensus control of multi-agent systems with general linear agent dynamics is investigated. A neighbor-based adaptive event-triggering strategy with a dynamic triggering threshold is proposed, which leads to a fully distributed control of the multi-agent system, depending only on the states of the neighboring agents at triggering moments. By using the Lyapunov method, we prove that the states of the agents converge asymptotically. In addition, the proposed event-triggering strategy is proven to exclude Zeno behavior. The numerical simulation results illustrate that the agent states achieve consensus in sense of asymptotic convergence. Furthermore, the proposed strategy is shown to be scalable in case of variable agent numbers.

## 1. Introduction

The recent research into multi-agent systems (MASs) paves the way for energy management and scheduling in smart grids [1], robot formation [2] and sensor networks [3,4]. Due to its wide applications, the consensus of MAS has attracted widespread attention [5,6]. As the size of MAS increases, the limits of the communication bandwidth and energy resources of the agents (such as mobile robots powered by batteries) have become difficult issues that need to be resolved in controller design. To deal with these problems, distributed communication was proposed by means of exchanging local information between neighboring agents [7]. Event-triggered strategies provide an effective solution in reducing the communication frequency and thus energy consumption of agents, by transmitting the state of agents only when the triggering condition is activated [7,8,9,10].

The cooperation control of MAS enables the organized agents to accomplish complex tasks. The research in this area attracts large attention for decades [11]. During this time, it evolves from centralized to distributed control [12]. Normally, centralized methods depend on massive communication, with which the bandwidth of communication of the MAS in large scale is heavily burdened. In addition, a high communication frequency leads to fast energy consumption, which shortens the usage time of the system powered by batteries. For these reasons, distributed control has been paid more attention. In most works of distributed control, the agents are assumed to communicate continuously. Furthermore, agents are aware of the topology of the communication network, such as in [13,14]. However, the continuous control requires the high-frequency communication of agents.

To overcome this drawback, sampled-data control is proposed, which utilizes time-triggered strategies with a predetermined sampling sequence. Recently, a large amount of works on sampled-data control was developed, such as [15,16,17]. In these works, agents need to communicate with their neighbors synchronously and select the appropriate sampling period, which is difficult to implement in practice.

The event-triggered control, which is more efficient than the sampled-data control in reducing unnecessary information transmission, is originally proposed in [18]. This method is then implemented in MASs in [19,20,21,22,23]. The critical issue of the event-triggered control is to determine the events and the triggering mechanism. The updates to the controller and exchanges of information occur exclusively when the triggering condition is satisfied. In general, the implementation of event-triggered strategies in MASs usually depends on the eigenvalues of the Laplacian matrix, which is global information associated with the communication graph, as shown in [24,25,26,27,28,29]. In order to improve the strategy, fully distributed event-triggered control was proposed very recently [30,31]. The consensus error is proven to be uniformly ultimately bounded.

Recently, adaptive control was successfully proposed in MASs. The agent with first-order dynamics in MASs was considered in [27]. Then, the control strategy was developed in agents with general linear agent dynamics [28]. Within this framework, more issues such as actuator and sensor faults were considered [32]. Considering bounded uncertainties, a static non-smooth event-triggered protocol is investigated in [29]. In [33], the external disturbances are considered. Notably, in the aforementioned papers, real-time feedback of the neighbor state is required.

To further reduce the frequency of communication between agents, dynamic event-triggered adaptive control was proposed recently [34,35], in which the event-triggered threshold was a dynamic variable. In [34], the dependence of eigenvalues of Laplacian matrix is mandatory. It is then released in [35], where the neighbors broadcast their information at the agents’ triggering instants. This increases the communication burden at triggering moments.

In this paper, we make significant modifications to the control strategy for MASs with a linear dynamic, which is proposed in [35]. Differently from the objective of the research in [23], where the consensus control of discrete multi-agent systems with parameter uncertainties was investigated, we focus on the adaptive event-triggered control for general linear agents in this paper. The main contributions are twofold. First, the proposed control strategy is independent on global information, and the consensus error converges asymptotically which is different from [31]. Second, continuously reading and listening to neighbor states is not required for any agents. Each agent exclusively broadcasts its information to neighbors when the event-triggered condition is satisfied. We also prove that the Zeno behavior can be excluded. The simulation results show that the consensus error converges asymptotically and the communication frequency is significantly decreased.

The rest of this paper is organized as follows. In Section 2, some preliminaries on graph theory are given. In Section 3, the adaptive event-triggered strategy is proposed. In Section 4, illustrative numerical simulation is carried out, which demonstrates the effectiveness of the theoretical results. Some convolutions are given in Section 5.

## 2. Preliminaries

### 2.1. Problem Statement

We consider a multi-agent system with *N* agents. Each agent is modeled by general linear dynamics as follows.

(1) $${\dot{x}}_{i}\left(t\right)=Ax_{i}\left(t\right)+Bu_{i}\left(t\right),i=1,2,\ldots ,N,$$

where $x_{i}\left(t\right)\in R^{n}$ and $u_{i}\left(t\right)\in R^{m}$ represent the state and control input of agent *i*, respectively. Matrices $A\in R^{n\times n}$ and $B\in R^{n\times m}$ are both constant matrices.

The objective of this paper is to design a fully distributed event-triggered consensus protocol for a leaderless network of the multi-agent system with agent dynamics modeled by (1). The requirements for this goal are as follows: the communication of the agent is distributed; each agent can only communicate with its neighboring agents and can only obtain their state information. The state variables of all agents ultimately reach asymptotic consensus (the definition of consensus in the multi-agent system is given later). Considering the constraints of communication network bandwidth and energy in real systems, it is also required that agents cannot communicate in real-time.

**Definition** **1** **([34]).**  *Consensus of Multi-Agent Systems. In a multi-agent system with N agents, for any given initial state,*

$$x\left(0\right)={\left[x_{1}^{T}\left(0\right),x_{2}^{T}\left(0\right),\ldots ,x_{N}^{T}\left(0\right)\right]}^{T},$$

*if $\lim_{t\to \infty }∥x_{i}\left(t\right)-x_{j}\left(t\right)∥=0$, where i,j=1,2,…,N, then the states of agents achieve consensus.*

### 2.2. Graph Theory

In a multi-agent system, the communication between agents can be modeled using graph theory. In the system, agents are represented by nodes, and the communication between agents is represented by edges. A graph G=(V,E), where V is a non-empty set of nodes in the graph, and E⊆V×V is a set of edges that do not intersect with the nodes. It is worth noting that an element in E is denoted by (i,j), which indicates that node *i* can send information to node *j*. In this case, node *i* is also called a neighbor of node *j*, and node *j* is an out-neighbor of node *i*. The set of neighbors of node *i* is denoted as $N_{i}=\left\{j:\left(j,i\right)\in E\right\}$, and the number of neighbors is defined as $|N_{i}|$. If, for any (i,j)∈E in the graph, there must exist (j,i)∈E, then the graph is called an undirected graph. In an undirected graph, if there exists a path (consisting of one or more edges) between every pair of distinct nodes, the undirected graph is connected; otherwise, it is not connected. For a graph G, the adjacency matrix is denoted by $A=\left[a_{ij}\right]\in R^{N\times N}$, where the elements are defined as $a_{ii}=0$, the off diagonal elements $a_{ij}=1$ if (i,j)∈E, or $a_{ij}=0$ otherwise. The Laplacian matrix associated with graph G is denoted by $L=\left[l_{ij}\right]\in R^{N\times N}$, where $l_{ii}=\sum_{j=1}^{N}a_{ij}$, $l_{ij}=-a_{ij}\left(i\neq j\right)$. Thus, $|N_{i}|=l_{ii}$. Two assumptions are given as follows.

**Assumption** **1.**  *The graph G is undirected and connected.*

**Assumption** **2.**  *(A, B) is stabilizable.*

According to Assumption A2, the algebraic Riccati equation $RA+A^{T}R-RBB^{T}R+I=0$ has a solution R>0 [35].

**Lemma** **1** **([31]).**  *The Laplacian matrix has a zero eigenvalue, and the corresponding eigenvector is $1_{n}$, which is a column vector with all elements equal to 1. Moreover, all non-zero eigenvalues of the Laplacian matrix have positive real parts. In an undirected graph, if the graph is connected, its corresponding Laplacian matrix L also has a single zero eigenvalue. The smallest non-zero eigenvalue of the matrix L, denoted as $\lambda_{2}\left(L\right)$, satisfies the equality $\lambda_{2}\left(L\right)=min_{x\neq 0,1^{T}x=0}\left(\frac{x^{T}Lx}{x^{T}x}\right)$.*

## 3. Main Results

**Figure 1:**
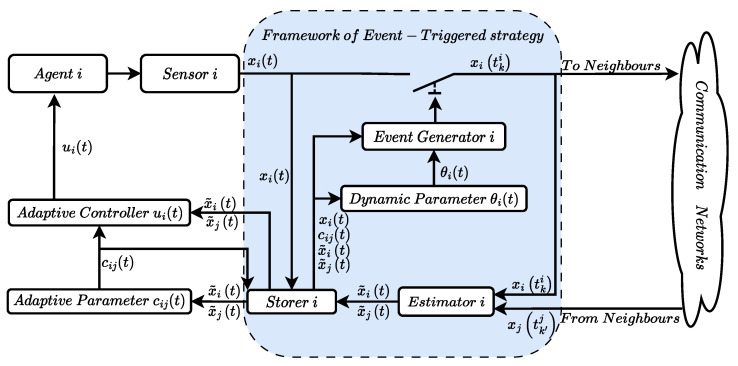
Event-triggered strategy framework for agent *i*.

In this section, an adaptive event-triggered consensus protocol is proposed. A schema of the controller is shown in Figure 1. In general, an event-based consensus protocol mainly consists of an event-based control law and a triggering function of the agent [30]. The controller design input and the triggering function are given in the sequel.

### 3.1. The Consensus Control Module

Inspired by paper [31], we propose the adaptive control input for agent *i* as follows.

(2) $$\left\{\begin{array}{cc} u_{i}\left(t\right) & =E\sum_{j=1}^{N}c_{ij}\left(t\right)a_{ij}\left({\hat{x}}_{i}\left(t\right)-{\hat{x}}_{j}\left(t\right)\right),\\ {\dot{c}}_{ij}\left(t\right) & ={\left({\hat{x}}_{i}\left(t\right)-{\hat{x}}_{j}\left(t\right)\right)}^{T}F\left({\hat{x}}_{i}\left(t\right)-{\hat{x}}_{j}\left(t\right)\right),i=1,\ldots ,N, \end{array}\right.$$

where $c_{ij}\left(0\right)\geq 0$. Matrices $E\in R^{m\times n}$ and $F=RBB^{T}R\in R^{n\times n}$ are feedback gains of the controller. The continuous state of agent *i* is estimated by the following equation.

(3) $$\begin{array}{cc} {\hat{x}}_{i}\left(t\right)=x_{i}\left(t_{k}^{i}\right)+g_{i}\left(t\right)\left(t-t_{k}^{i}\right), & \forall t\in [t_{k}^{i},t_{k+1}^{i}) \end{array}$$

where

$$g\left(t\right)=\left\{\begin{array}{cc} 0, & t\leq t_{2}^{i}\\ \frac{x_{i}\left(t_{k}^{i}\right)-x_{i}\left(t_{k-1}^{i}\right)}{t_{k}^{i}-t_{k-1}^{i}}, & t>t_{2}^{i} \end{array}\right.$$

Note that $x_{i}\left(t_{1}^{i}\right)=x_{i}\left(0\right)$.

**Remark** **1.**  *In (2), the adaptive parameter $c_{ij}\left(t\right)$ is used to regulate the weights of communication links on the topology. The protocol of $c_{ij}\left(t\right)$ is designed only based on the state variables of agent i and j at triggering moments. Therefore, the controller is fully distributed. In addition, the continuous measuring and listening of agent states are avoided.*

**Remark** **2.**  *If $c_{ij}\left(0\right)\geq 0$, we conclude that $c_{ij}\left(t\right)$ is always greater than zero, since ${\dot{c}}_{ij}\geq 0$.*

### 3.2. The Event-Triggering Protocol

The triggering function is designed as follows:

(4) $$\left\{\begin{array}{c} f_{i}\left(t\right)=\sum_{j=1}^{N}\left(1+c_{ij}\right)a_{ij}e_{i}^{T}Fe_{i}-\frac{1}{8}\sum_{j=1}^{N}a_{ij}{\left({\hat{x}}_{i}-{\hat{x}}_{j}\right)}^{T}F\left({\hat{x}}_{i}-{\hat{x}}_{j}\right)-\theta_{i},\\ {\dot{\theta }}_{i}=-\rho_{i}\theta_{i}-\sigma_{i}\left[\sum_{j=1}^{N}\left(1+c_{ij}\right)a_{ij}e_{i}^{T}Fe_{i}-\frac{1}{8}\sum_{j=1}^{N}a_{ij}{\left({\hat{x}}_{i}-{\hat{x}}_{j}\right)}^{T}F\left({\hat{x}}_{i}-{\hat{x}}_{j}\right)\right], \end{array}\right.$$

where $e_{i}\left(t\right)≜{\hat{x}}_{i}\left(t\right)-x_{i}\left(t\right),i=1,\ldots ,N$ is the estimating error of the state of agent *i*. Parameters $\rho_{i}$ and $\sigma_{i}$ are both positive constant scalars, which are not equal for agents. The selection of these parameters is discussed later.

The triggering time for agent *i* is defined by $t_{k+1}^{i}≜\inf\left\{t>t_{k}^{i}|f_{i}\left(t\right)\geq 0\right\}$, where $t_{k}^{i}$ represents the *k*-th triggering time of agent *i*, and $f_{i}\left(t\right)\geq 0$ is called the event trigger condition. Note that $t_{1}^{i}=0$. As can be seen from the event-based consensus framework in Figure 1, the update of the controller signal of agent *i* depends on its own states at its triggering moments and the states of its neighbors at their latest triggering moments.

In the second equation in (4), by choosing some positive scalar $\rho_{i}$ and $\sigma_{i}$, we can obtain ${\dot{\theta }}_{i}>-\left(\rho_{i}+\sigma_{i}\right)\theta_{i}$ except in the triggering moments. According to the comparison principle, it holds that $\theta_{i}\left(t\right)>\theta_{i}\left(0\right)\exp\left[-\left(\rho_{i}+\sigma_{i}\right)t\right]$.

### 3.3. Consensus Analysis

In the sequel, we will prove that the multi-agent system achieves asymptotic consensus under the proposed protocol on avoiding Zeno behavior. Let us define by $\xi_{i}≜x_{i}-\left(1/N\right)\sum_{j=1}^{N}x_{j}$ the *consensus error* of agent *i*. The compact form of $\xi_{i}$ for all the agents is represented by vector $\xi^{T}=\left[\xi_{1},\cdots ,\xi_{N}\right]$.

**Theorem** **1.**  *With Assumptions 1 and 2 and choosing $E=-B^{T}R$, where R>0 is the solution of the algebraic Riccati equation (ARE) $RA+A^{T}R-RBB^{T}R+I=0$, then, the consensus error ξ ultimately achieves asymptotic consensus and the adaptive parameter $c_{ij}$ in Equation (2) is uniformly ultimately bounded, if the adaptive protocol (2) satisfies $c_{ij}\left(0\right)\geq 0$, and the event-triggering function (4) satisfies $\theta_{i}\left(0\right)>0$, $\sigma_{i}<1$, and $\sigma_{i}+\rho_{i}>1$.*

**Proof.**  Based on the design of the control input $u_{i}\left(t\right)=E\sum_{j=1}^{N}c_{ij}\left(t\right)a_{ij}\left({\hat{x}}_{i}\left(t\right)-{\hat{x}}_{j}\left(t\right)\right)$ and the ARE, we chose the feedback matrix in the control input as $E=-B^{T}R$ and the Lyapunov function candidate as follows:

(5) $$\begin{array}{c} V_{1}=\sum_{i=1}^{N}\xi_{i}^{T}R\xi_{i}, \end{array}$$

Its time derivative yields $\dot{\xi_{i}}=A\xi_{i}+BE\sum_{j=1}^{N}c_{ij}\left(t\right)a_{ij}\left({\hat{x}}_{i}-{\hat{x}}_{j}\right)$, and $\xi_{i}-\xi_{j}=x_{i}-x_{j}$.

Then, the derivative of $V_{1}$ yields:

(6) $$\begin{array}{cc} {\dot{V}}_{1}= & 2\sum_{i=1}^{N}\xi_{i}^{T}R\dot{\xi_{i}}\\ = & \sum_{i=1}^{N}\xi_{i}^{T}\left(RA+A^{T}R\right)\xi_{i}+2\sum_{i=1}^{N}\xi_{i}^{T}RBE\sum_{j=1}^{N}c_{ij}a_{ij}\left({\hat{x}}_{i}-{\hat{x}}_{j}\right). \end{array}$$

Since we assume that the communication topology network of MAS in this paper is undirected. The entries in the adjacency matrix satisfy $a_{ij}=a_{ji}=1$. Then, the adaptive gains are given such that $c_{ij}\left(t\right)=c_{ji}\left(t\right)$. Due to $E=-B^{T}R,F=RBB^{T}R$, we have

(7) $$\begin{array}{c} \sum_{i=1}^{N}\xi_{i}^{T}RBE\sum_{j=1}^{N}c_{ij}a_{ij}\left({\hat{x}}_{i}-{\hat{x}}_{j}\right)=-\frac{1}{2}\sum_{i=1}^{N}\sum_{j=1}^{N}c_{ij}a_{ij}{\left(\xi_{i}-\xi_{j}\right)}^{T}F\left({\hat{x}}_{i}-{\hat{x}}_{j}\right). \end{array}$$

By substituting Equation (7) in (6), we can rewrite ${\dot{V}}_{1}$ as follows.

(8) $$\begin{array}{c} {\dot{V}}_{1}=\sum_{i=1}^{N}\xi_{i}^{T}\left(RA+A^{T}R\right)\xi_{i}-\sum_{i=1}^{N}\sum_{j=1}^{N}c_{ij}a_{ij}{\left(\xi_{i}-\xi_{j}\right)}^{T}F\left({\hat{x}}_{i}-{\hat{x}}_{j}\right). \end{array}$$

Recalling that $\xi_{i}-\xi_{j}=x_{i}-x_{j}$ and $e_{i}={\hat{x}}_{i}-x_{i}$, we obtain the following equation.

(9) $$\xi_{i}-\xi_{j}=\left({\hat{x}}_{i}-{\hat{x}}_{j}\right)-\left(e_{i}-e_{j}\right).$$

According to (9), we can rewrite (8) as follows.

(10) $$\begin{array}{cc} {\dot{V}}_{1}= & \sum_{i=1}^{N}\xi_{i}^{T}\left(RA+A^{T}R\right)\xi_{i}-\sum_{i=1}^{N}\sum_{j=1}^{N}c_{ij}a_{ij}{\left({\hat{x}}_{i}-{\hat{x}}_{j}\right)}^{T}F\left({\hat{x}}_{i}-{\hat{x}}_{j}\right)\\ \; & +\sum_{i=1}^{N}\sum_{j=1}^{N}c_{ij}a_{ij}{\left(e_{i}-e_{j}\right)}^{T}F\left({\hat{x}}_{i}-{\hat{x}}_{j}\right). \end{array}$$

Using Young’s inequality [31], we can obtain

(11) $$\begin{array}{cc} \; & \sum_{i=1}^{N}\sum_{j=1}^{N}c_{ij}a_{ij}{\left(e_{i}-e_{j}\right)}^{T}F\left({\hat{x}}_{i}-{\hat{x}}_{j}\right)\\ \; & \leq \frac{1}{4}\sum_{i=1}^{N}\sum_{j=1}^{N}c_{ij}a_{ij}{\left({\hat{x}}_{i}-{\hat{x}}_{j}\right)}^{T}F\left({\hat{x}}_{i}-{\hat{x}}_{j}\right)+\sum_{i=1}^{N}\sum_{j=1}^{N}c_{ij}a_{ij}{\left(e_{i}-e_{j}\right)}^{T}F\left(e_{i}-e_{j}\right). \end{array}$$

Substituting (11) into (10), we can rewrite ${\dot{V}}_{1}$ as follows.

(12) $$\begin{array}{cc} {\dot{V}}_{1}\leq & \sum_{i=1}^{N}\xi_{i}^{T}\left(RA+A^{T}R\right)\xi_{i}-\frac{3}{4}\sum_{i=1}^{N}\sum_{j=1}^{N}c_{ij}a_{ij}{\left({\hat{x}}_{i}-{\hat{x}}_{j}\right)}^{T}F\left({\hat{x}}_{i}-{\hat{x}}_{j}\right)\\ \; & +\sum_{i=1}^{N}\sum_{j=1}^{N}c_{ij}a_{ij}{\left(e_{i}-e_{j}\right)}^{T}F\left(e_{i}-e_{j}\right). \end{array}$$

To utilize the algebraic Riccati equation (ARE), we add a second term $V_{2}$ into (5), which is given as follows.

(13) $$V_{1}+V_{2}=\sum_{i=1}^{N}\xi_{i}^{T}R\xi_{i}+\sum_{i=1}^{N}\sum_{j=1}^{N}\frac{3a_{ij}{\left(c_{ij}-\alpha \right)}^{2}}{8}$$

where α is a positive constant. Recall that ${\dot{c}}_{ij}\left(t\right)={\left({\hat{x}}_{i}\left(t\right)-{\hat{x}}_{j}\left(t\right)\right)}^{T}F\left({\hat{x}}_{i}\left(t\right)-{\hat{x}}_{j}\left(t\right)\right),i=1,\ldots ,N$; the time derivative of Equation (13) yields

(14) $$\begin{array}{cc} {\dot{V}}_{1}+{\dot{V}}_{2}\leq & \sum_{i=1}^{N}\xi_{i}^{T}\left(RA+A^{T}R\right)\xi_{i}-\frac{3}{4}\sum_{i=1}^{N}\sum_{j=1}^{N}\alpha a_{ij}{\left({\hat{x}}_{i}-{\hat{x}}_{j}\right)}^{T}F\left({\hat{x}}_{i}-{\hat{x}}_{j}\right)\\ \; & +\sum_{i=1}^{N}\sum_{j=1}^{N}c_{ij}a_{ij}{\left(e_{i}-e_{j}\right)}^{T}F\left(e_{i}-e_{j}\right) \end{array}.$$

Note that

(15) $$\begin{array}{cc} \; & -\sum_{i=1}^{N}\sum_{j=1}^{N}a_{ij}{\left({\hat{x}}_{i}-{\hat{x}}_{j}\right)}^{T}F\left({\hat{x}}_{i}-{\hat{x}}_{j}\right)=-\left(\sum_{i=1}^{N}\sum_{j=1}^{N}a_{ij}{\left(\xi_{i}-\xi_{j}\right)}^{T}F\left(\xi_{i}-\xi_{j}\right)\right.\\ \; & \left.+\sum_{i=1}^{N}\sum_{j=1}^{N}a_{ij}{\left(e_{i}-e_{j}\right)}^{T}F\left(e_{i}-e_{j}\right)+2\sum_{i=1}^{N}\sum_{j=1}^{N}a_{ij}{\left(x_{i}-x_{j}\right)}^{T}F\left(e_{i}-e_{j}\right)\right), \end{array}$$

where

(16) $$\begin{array}{cc} -\sum_{i=1}^{N}\sum_{j=1}^{N}a_{ij}{\left(x_{i}-x_{j}\right)}^{T}F\left(e_{i}-e_{j}\right)\leq & \frac{1}{4}\sum_{i=1}^{N}\sum_{j=1}^{N}a_{ij}{\left(x_{i}-x_{j}\right)}^{T}F\left(x_{i}-x_{j}\right)\\ \; & +\sum_{i=1}^{N}\sum_{j=1}^{N}a_{ij}{\left(e_{i}-e_{j}\right)}^{T}F\left(e_{i}-e_{j}\right), \end{array}$$

Combining (15) and (16), we can obtain

(17) $$\begin{array}{cc} -\sum_{i=1}^{N}\sum_{j=1}^{N}a_{ij}{\left({\hat{x}}_{i}-{\hat{x}}_{j}\right)}^{T}F\left({\hat{x}}_{i}-{\hat{x}}_{j}\right)\leq & -\frac{1}{2}\sum_{i=1}^{N}\sum_{j=1}^{N}a_{ij}{\left(\xi_{i}-\xi_{j}\right)}^{T}F\left(\xi_{i}-\xi_{j}\right)\\ \; & +\sum_{i=1}^{N}\sum_{j=1}^{N}a_{ij}{\left(e_{i}-e_{j}\right)}^{T}F\left(e_{i}-e_{j}\right). \end{array}$$

Substituting (17) into (14), we have

(18) $$\begin{array}{cc} {\dot{V}}_{1}+{\dot{V}}_{2}\leq & \sum_{i=1}^{N}\xi_{i}^{T}\left(RA+A^{T}R\right)\xi_{i}-\frac{\alpha }{4}\sum_{i=1}^{N}\sum_{j=1}^{N}a_{ij}{\left(\xi_{i}-\xi_{j}\right)}^{T}F\left(\xi_{i}-\xi_{j}\right)\\ \; & +\frac{\alpha }{2}\sum_{i=1}^{N}\sum_{j=1}^{N}a_{ij}{\left(e_{i}-e_{j}\right)}^{T}F\left(e_{i}-e_{j}\right)\\ \; & -\frac{1}{4}\sum_{i=1}^{N}\sum_{j=1}^{N}\alpha a_{ij}{\left({\hat{x}}_{i}-{\hat{x}}_{j}\right)}^{T}F\left({\hat{x}}_{i}-{\hat{x}}_{j}\right)\\ \; & +\sum_{i=1}^{N}\sum_{j=1}^{N}c_{ij}a_{ij}{\left(e_{i}-e_{j}\right)}^{T}F\left(e_{i}-e_{j}\right). \end{array}$$

We rewrite Equation (18) as follows.

(19) $$\begin{array}{cc} \; & {\dot{V}}_{1}+{\dot{V}}_{2}\leq \xi^{T}\left[I_{N}⊗\left(RA+A^{T}R\right)-\frac{\alpha }{4}L⊗F\right]\xi \\ \; & +\frac{\alpha }{2}\sum_{i=1}^{N}\left[\sum_{j=1}^{N}\left(1+\frac{2}{\alpha }\cdot c_{ij}\right)a_{ij}{\left(e_{i}-e_{j}\right)}^{T}F\left(e_{i}-e_{j}\right)-\frac{1}{2}\sum_{j=1}^{N}a_{ij}{\left({\hat{x}}_{i}-{\hat{x}}_{j}\right)}^{T}F\left({\hat{x}}_{i}-{\hat{x}}_{j}\right)\right], \end{array}$$

where $\xi ={\left[\xi_{1}^{T},\ldots ,\xi_{N}^{T}\right]}^{T}$.

Note that

(20) $$\begin{array}{cc} \sum_{i=1}^{N}\sum_{j=1}^{N}c_{ij}a_{ij}{\left(e_{i}-e_{j}\right)}^{T}F\left(e_{i}-e_{j}\right)\leq & 2\sum_{i=1}^{N}\sum_{j=1}^{N}c_{ij}a_{ij}e_{i}^{T}Fe_{i}+2\sum_{i=1}^{N}\sum_{j=1}^{N}c_{ij}a_{ij}e_{j}^{T}Fe_{j}\\ \; & =4\sum_{i=1}^{N}\sum_{j=1}^{N}c_{ij}a_{ij}e_{i}^{T}Fe_{i}. \end{array}$$

Substituting (20) into (19), we obtain

(21) $$\begin{array}{cc} {\dot{V}}_{1}+{\dot{V}}_{2}\leq & \xi^{T}\left[I_{N}⊗\left(RA+A^{T}R\right)-\frac{\alpha }{4}L⊗F\right]\xi \\ \; & +2\alpha \sum_{i=1}^{N}\left[\sum_{j=1}^{N}\left(1+\frac{2}{\alpha }\cdot c_{ij}\right)a_{ij}e_{i}^{T}Fe_{i}-\frac{1}{8}\sum_{j=1}^{N}a_{ij}{\left({\hat{x}}_{i}-{\hat{x}}_{j}\right)}^{T}F\left({\hat{x}}_{i}-{\hat{x}}_{j}\right)\right]. \end{array}$$

According to Lemma 1, we have $\xi^{T}\left(L⊗F\right)\xi \geq \lambda_{2}\left(L\right)\xi^{T}\left(I_{N}⊗F\right)\xi$. Then, we can rewrite (21) as follows.

(22) $$\begin{array}{cc} {\dot{V}}_{1}+{\dot{V}}_{2}\leq & \xi^{T}\left[I_{N}⊗\left(RA+A^{T}R\right)-\frac{\lambda_{2}\left(L\right)\alpha }{4}I_{N}⊗F\right]\xi \\ \; & +2\alpha \sum_{i=1}^{N}\left[\sum_{j=1}^{N}\left(1+\frac{2}{\alpha }\cdot c_{ij}\right)a_{ij}e_{i}^{T}Fe_{i}-\frac{1}{8}\sum_{j=1}^{N}a_{ij}{\left({\hat{x}}_{i}-{\hat{x}}_{j}\right)}^{T}F\left({\hat{x}}_{i}-{\hat{x}}_{j}\right)\right], \end{array}$$

where α satisfies $\alpha \geq \max\{2,4/\lambda_{2}\left(L\right)\}$. Then,

(23) $$\begin{array}{cc} {\dot{V}}_{1}+{\dot{V}}_{2}\leq & \xi^{T}\left[I_{N}⊗\left(RA+A^{T}R\right)-I_{N}⊗F\right]\xi \\ \; & +2\alpha \sum_{i=1}^{N}\left[\sum_{j=1}^{N}\left(1+c_{ij}\right)a_{ij}e_{i}^{T}Fe_{i}-\frac{1}{8}\sum_{j=1}^{N}a_{ij}{\left({\hat{x}}_{i}-{\hat{x}}_{j}\right)}^{T}F\left({\hat{x}}_{i}-{\hat{x}}_{j}\right)\right]\\ \leq & -\xi^{T}\xi +2\alpha \sum_{i=1}^{N}\left[\sum_{j=1}^{N}\left(1+c_{ij}\right)a_{ij}e_{i}^{T}Fe_{i}-\frac{1}{8}\sum_{j=1}^{N}a_{ij}{\left({\hat{x}}_{i}-{\hat{x}}_{j}\right)}^{T}F\left({\hat{x}}_{i}-{\hat{x}}_{j}\right)\right]. \end{array}$$

We now reconstruct the Lyapunov function by adding a third term $V_{3}=2\alpha \sum_{i=1}^{N}\theta_{i}$ into (13), as follows.

(24) $$V_{1}+V_{2}+V_{3}=\sum_{i=1}^{N}\xi_{i}^{T}R\xi_{i}+\sum_{i=1}^{N}\sum_{j=1}^{N}\frac{3a_{ij}{\left(c_{ij}-\alpha \right)}^{2}}{8}+2\alpha \sum_{i=1}^{N}\theta_{i}$$

where $\theta_{i}\left(0\right)>0$. We calculate the derivative of (24), with $h_{i}=\sum_{j=1}^{N}\left(1+c_{ij}\right)a_{ij}e_{i}^{T}Fe_{i}-\frac{1}{8}\sum_{j=1}^{N}a_{ij}{\left({\hat{x}}_{i}-{\hat{x}}_{j}\right)}^{T}F\left({\hat{x}}_{i}-{\hat{x}}_{j}\right)$, which yields

(25) $$\begin{array}{c} {\dot{V}}_{1}+{\dot{V}}_{2}+{\dot{V}}_{3}\leq -\xi^{T}\xi +2\alpha \sum_{i=1}^{N}\left[-\rho_{i}\theta_{i}+\left(1-\sigma_{i}\right)h_{i}\right], \end{array}$$

where $\sigma_{i}<1$. Considering (4), the inequality (25) yields

(26) $$\begin{array}{c} {\dot{V}}_{1}+{\dot{V}}_{2}+{\dot{V}}_{3}\leq -\xi^{T}\xi +2\alpha \sum_{i=1}^{N}\left[\left(1-\sigma_{i}-\rho_{i}\right)\theta_{i}\right], \end{array}$$

By choosing $\sigma_{i}+\rho_{i}>1$, we have ${\dot{V}}_{1}+{\dot{V}}_{2}+{\dot{V}}_{3}\leq 0$. The Lyapunov function is equal to zero if and only if ξ=0 and θ=0. Hence, the largest invariant set is composed by Ω={ξ=0,θ=0}, where $\theta ={\left[\theta_{1},\cdots ,\theta_{n}\right]}^{T}$. By using LaSalle’s invariance principle, we conclude that ξ(t)→0, θ(t)→0 as t→∞ and $c_{ij}\leq \bar{c}$ where $\bar{c}$ is a limited positive constant. Thus, the consensus of (1) is achieved in the sense of asymptotic convergence. That ends the proof. □

In the sequel, we will discuss how the Zeno behavior is excluded. Let us define $\Delta_{k}^{i}=t_{k+1}^{i}-t_{k}^{i}$.

**Theorem** **2.**  *If the parameters of the controller (2) and triggering function (4) are selected satisfying $c_{ij}\left(0\right)\geq 0$, $\theta_{i}\left(0\right)>0$, $\sigma_{i}<1$, and $\sigma_{i}+\rho_{i}>1$, then, there does not exist any positive finite constant scalars T, which makes $\lim_{m\to \infty }\sum_{k=0}^{m}\Delta_{k}^{i}<T$.*

**Proof.**  Recall that $e_{i}\left(t\right)≜{\hat{x}}_{i}\left(t\right)-x_{i}\left(t\right),i=1,\ldots ,N$; then,

(27) $${\dot{e}}_{i}=g_{i}\left(t\right)-\left(Ax_{i}+\sum_{j=1}^{N}c_{ij}a_{ij}BE\left({\hat{x}}_{i}-{\hat{x}}_{j}\right)\right).$$

The derivative of $∥e_{i}\left(t\right)∥$ yields

(28) $$\begin{array}{cc} \frac{d∥e_{i}\left(t\right)∥}{dt} & =\frac{e_{i}^{T}}{∥e_{i}∥}{\dot{e}}_{i}\leq ∥{\dot{e}}_{i}∥\leq ∥A∥∥e_{i}∥+∥g_{i}∥+∥Ax_{i}+\sum_{j=1}^{N}c_{ij}a_{ij}BE\left({\hat{x}}_{i}-{\hat{x}}_{j}\right)∥. \end{array}$$

where $t\in [t_{k}^{i},t_{k+1}^{i})$. According to theorem 1, we infer that $∥\dot{x}∥=∥Ax_{i}+\sum_{j=1}^{N}c_{ij}a_{ij}BE\left({\hat{x}}_{i}-{\hat{x}}_{j}\right)∥$ is bounded. Thus, ${\bar{\omega }}_{i}$ satisfies ${\bar{\omega }}_{i}>∥g_{i}∥+∥Ax_{i}+\sum_{j=1}^{N}c_{ij}a_{ij}BE\left({\hat{x}}_{i}-{\hat{x}}_{j}\right)∥$.

We process the proof by contradiction in the sequel. We first assume that the Zeno-behavior exists, such that

(29) $$\lim_{k\to \infty }t_{k}^{i}=\lim_{k\to \infty }t_{k+1}^{i}$$

a. When ∥A∥≠0,
  By using the comparison principle, we can rewrite (28) as follows.
  $$\begin{array}{cc} ∥e_{i}\left(t\right)∥\leq \frac{{\bar{\omega }}_{i}}{∥A∥}\left(e^{∥A∥(t-t_{k}^{i})}-1\right), & t\in [t_{k}^{i},t_{k+1}^{i}) \end{array}$$
  Therefore,
  (30) $$\begin{array}{cc} ∥e_{i}^{T}\left(t\right)RB∥\leq \frac{({\bar{\omega }}_{i})∥RB∥}{∥A∥}\left(e^{∥A∥(t-t_{k}^{i})}-1\right), & t\in [t_{k}^{i},t_{k+1}^{i}). \end{array}$$
  According to (4), we can infer that at triggering instant $t=t_{k+1}^{i}$,
  $$\begin{array}{cc} & \sum_{j=1}^{N}\left(1+c_{ij}\left(t_{k+1}^{i}\right)\right)a_{ij}e_{i}{\left(t_{k+1}^{i}\right)}^{T}Fe_{i}\left(t_{k+1}^{i}\right)\\ & -\frac{1}{8}\sum_{j=1}^{N}a_{ij}{\left({\hat{x}}_{i}\left(t_{k+1}^{i}\right)-{\hat{x}}_{j}\left(t_{k+1}^{i}\right)\right)}^{T}F\left({\hat{x}}_{i}\left(t_{k+1}^{i}\right)-{\hat{x}}_{j}\left(t_{k+1}^{i}\right)\right)\\ & \geq \theta_{i}\left(0\right)e^{-\left(\rho_{i}+\sigma_{i}\right)t_{k+1}^{i}}, \end{array}$$
  Then,
  (31) $$\begin{array}{c} ∥e_{i}^{T}\left(t_{k+1}^{i}\right){RB∥}^{2}\geq \frac{\frac{1}{8}\sum_{j=1}^{N}{∥q_{j}^{T}\left(t_{k+1}^{i}\right)RB∥}^{2}+\theta_{i}\left(0\right)e^{-\left(\rho_{i}+\sigma_{i}\right)t_{k+1}^{i}}}{l_{ii}\left(1+\bar{c}\right)}. \end{array}$$
  where $q_{j}\left(t_{k+1}^{i}\right)={\hat{x}}_{i}\left(t_{k+1}^{i}\right)-{\hat{x}}_{j}\left(t_{k+1}^{i}\right)$.
  According to (29), we have
  (32) $$\frac{\frac{1}{8}\sum_{j=1}^{N}{∥q_{j}^{T}\left(t_{k+1}^{i}\right)RB∥}^{2}+\theta_{i}\left(0\right)e^{-\left(\rho_{i}+\sigma_{i}\right)t_{k+1}^{i}}}{l_{ii}\left(1+\bar{c}\right)}\leq {\left(\frac{{\bar{\omega }}_{i}∥RB∥}{∥A∥}\left(e^{∥A∥(t_{k+1}^{i}-t_{k}^{i})}-1\right)\right)}^{2}$$
  which infers that $\theta_{i}\left(0\right)<0$. It contradicts the condition $\theta_{i}\left(0\right)>0$. Therefore, Equation (29) is not true, i.e., the Zeno behavior does not exist.
b. When ∥A∥=0, according to Equations (28) and (29), Equation (31) yields,
  (33) $$\frac{\frac{1}{8}\sum_{j=1}^{N}{∥q_{j}^{T}\left(t_{k+1}^{i}\right)RB∥}^{2}+\theta_{i}\left(0\right)e^{-\left(\rho_{i}+\sigma_{i}\right)t_{k+1}^{i}}}{d_{i}\left(1+\bar{c}\right)}\leq \left({\bar{\omega }}_{i}\right)∥RB∥\left(t_{k+1}^{i}-t_{k}^{i}\right),t\in \left[t_{k}^{i},t_{k+1}^{i}\right).$$
  which also infers that $\theta_{i}\left(0\right)<0$. It contradicts the condition $\theta_{i}\left(0\right)>0$. Therefore, Equation (29) is not true, i.e., the Zeno behavior does not exist.

According to the aforementioned analysis, the Zeno behavior of MAS is excluded. That ends the proof. □

## 4. Simulation Results

In this section, we carry out the numerical simulation to validate the proposed theoretical result and compare it to some related works to show the improvements.

We consider the following two scenarios. In the first scenario, a multi-agent system with five agents is considered. The dynamics of each agent are modeled by a third-order linear system (34).

(34) $$\left.A=\left[\begin{array}{ccc} 0 & 1 & 0\\ 0 & 0 & 1\\ 0 & 0 & 0 \end{array}\right.\right],B=\left[\begin{array}{c} 0\\ 0\\ 1 \end{array}\right]$$

The initial states of the five agents are given by $x_{1}\left(0\right)={\left[0.25,0.25,0.25\right]}^{T}$, $x_{2}\left(0\right)={\left[1.25,1.25,1.25\right]}^{T}$, $x_{3}\left(0\right)={\left[2,2,2\right]}^{T}$, $x_{4}\left(0\right)={\left[2.5,2.5,2.5\right]}^{T}$ and $x_{5}\left(0\right)={\left[4,4,4\right]}^{T}$.

**Figure 2:**
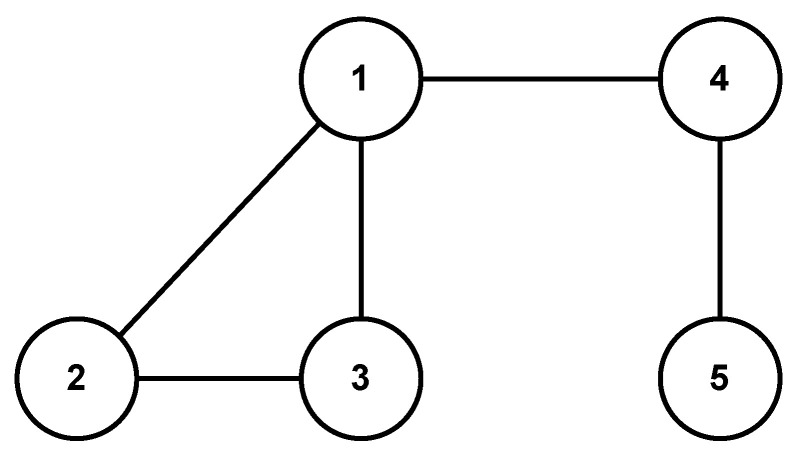
Undirected network topology of the MASs with five agents.

The communication topology of the MAS is shown in Figure 2. It is evident that the graph is undirected and connected, as its Laplacian yields

$$L=\left[\begin{array}{ccccc} 3 & -1 & -1 & -1 & 0\\ -1 & 2 & -1 & 0 & 0\\ -1 & -1 & 2 & 0 & 0\\ -1 & 0 & 0 & 2 & -1\\ 0 & 0 & 0 & -1 & 1 \end{array}\right].$$

In controller (2), we choose $c_{ij}\left(0\right)=0.25$, if $j\in N_{i}$. The gain matrices are obtained by solving the ARE $RA+A^{T}R-RBB^{T}R+I=0$, as follows.

$$E=\left[\begin{array}{c} -1.000\;-2.4142\;-2.4142 \end{array}\right],F=\left[\begin{array}{ccc} 1.0000 & 2.4142 & 2.4142\\ 2.4142 & 5.8284 & 5.8284\\ 2.4142 & 5.8284 & 5.8284 \end{array}\right].$$

In (4), the initial value of the event-triggered threshold is assigned by $\theta_{i}\left(0\right)=0.25$. The scalars $\rho_{i}$ and $\sigma_{i}$ are given by $\rho_{i}=0.3,\sigma_{i}=0.8$, where i∈V.

For the sake of comparison, we, respectively, carried out the simulation using the control strategies proposed in [31,35]. In both simulations, the agent model and initial states are given the same as in (34). In [31], the adaptive parameters are also initialized by $c_{ij}\left(0\right)=0.25$, if $j\in N_{i}$. In [35], the parameters of the triggering threshold are given by $\rho_{i}=0.3,\sigma_{i}=0.8$, where i∈V as well.

In the second scenario, we consider a MAS with eight agents. The dynamic of each agent is modeled as follows.

(35) $$\left.A=\left[\begin{array}{ccc} 0 & 1 & 0\\ 0 & 0 & 1\\ 0 & -0.4 & -0.5 \end{array}\right.\right],B=\left[\begin{array}{c} 0\\ 0\\ 1 \end{array}\right]$$

The initial states of the eight agents are given by $x_{1}\left(0\right)={\left[0.25,0.25,0.25\right]}^{T}$, $x_{2}\left(0\right)={\left[1.5,1.5,1.5\right]}^{T}$, $x_{3}\left(0\right)={\left[1.25,1.25,1.25\right]}^{T}$, $x_{4}\left(0\right)={\left[2,2,2\right]}^{T}$, $x_{5}\left(0\right)={\left[2.5,2.5,2.5\right]}^{T}$, $x_{6}\left(0\right)={\left[3.25,3.25,3.25\right]}^{T}$, $x_{7}\left(0\right)={\left[4.5,4.5,4.5\right]}^{T}$ and $x_{8}\left(0\right)={\left[4.75,4.75,4.75\right]}^{T}$.

**Figure 3:**
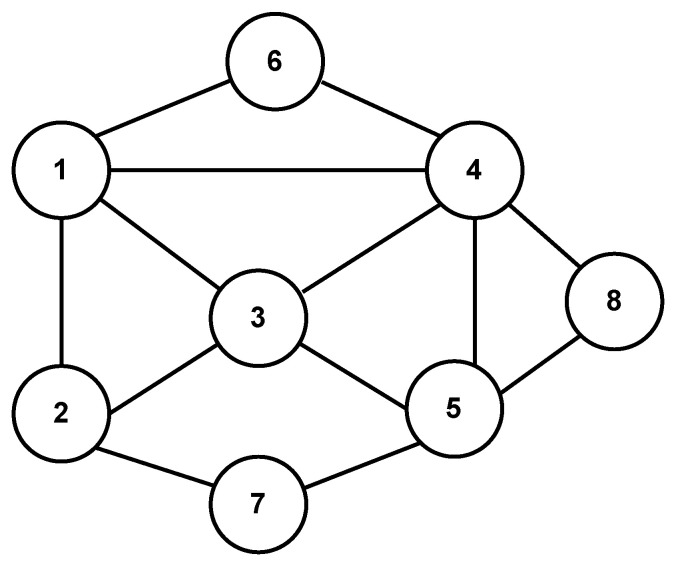
Undirected network topology of the MASs with eight agents.

The network topology of the MAS with eight agents is shown in Figure 3. The graph also is undirected and connected, and its Laplacian matrix is as follows.

$$L=\left[\begin{array}{cccccccc} 4 & -1 & -1 & -1 & 0 & -1 & 0 & 0\\ -1 & 3 & -1 & 0 & 0 & 0 & -1 & 0\\ -1 & -1 & 4 & -1 & -1 & 0 & 0 & 0\\ -1 & 0 & -1 & 5 & -1 & -1 & 0 & -1\\ 0 & 0 & -1 & -1 & 4 & 0 & -1 & -1\\ -1 & 0 & 0 & -1 & 0 & 2 & 0 & 0\\ 0 & -1 & 0 & 0 & -1 & 0 & 2 & 0\\ 0 & 0 & 0 & -1 & -1 & 0 & 0 & 2 \end{array}\right].$$

In controller (2), we choose $c_{ij}\left(0\right)=0.25$, if $j\in N_{i}$. The gain matrices are obtained by solving the ARE $RA+A^{T}R-RBB^{T}R+I=0$, as follows.

$$E=\left[\begin{array}{c} -1\;-1.9956\;-1.7893 \end{array}\right],F=\left[\begin{array}{ccc} 1 & 1.9956 & 1.7893\\ 1.9956 & 3.9823 & 3.5707\\ 1.7893 & 3.5707 & 3.2018 \end{array}\right].$$

In (4), the initial value of the event-triggered threshold is assigned by $\theta_{i}\left(0\right)=0.25$. The scalars $\rho_{i}$ and $\sigma_{i}$ are given by $\rho_{i}=0.3,\sigma_{i}=0.8$, where i∈V.

For the sake of comparison, we, respectively, carried out the simulation using the control strategies proposed in [31,35]. In both simulations, the agent model and initial states are given the same as in (35). In [31], the adaptive parameters are also initialized by $c_{ij}\left(0\right)=0.25$, if $j\in N_{i}$. In [35], the parameters of the triggering threshold are given by $\rho_{i}=0.3,\sigma_{i}=0.8$, where i∈V as well.

**Figure 4:**
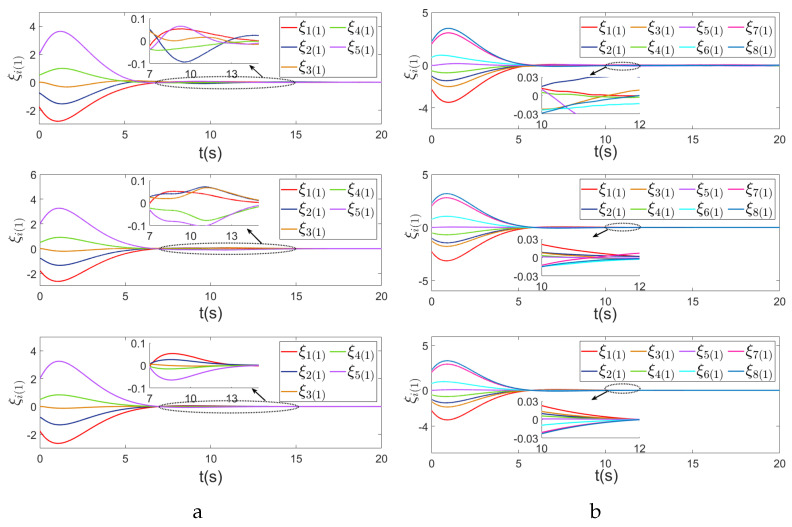
First components of consensus errors. (**a**) First components of consensus errors of five agents $\xi_{i(1)}$, i∈V, using the control strategies in [31,35] and this paper respectively. (**b**) First components of consensus errors of eight agents $\xi_{i(1)}$, i∈V, using the control strategies in [31,35] and this paper respectively.

Using the controllers proposed in this paper and in [31,35], the comparison of the first components $\xi_{i(1)}$, i∈V of consensus error ξ is given in Figure 4, in which we observe that by using the proposed control, the MAS achieves consensus asymptotically and the convergence speed is faster than that in [31,35].

**Figure 5:**
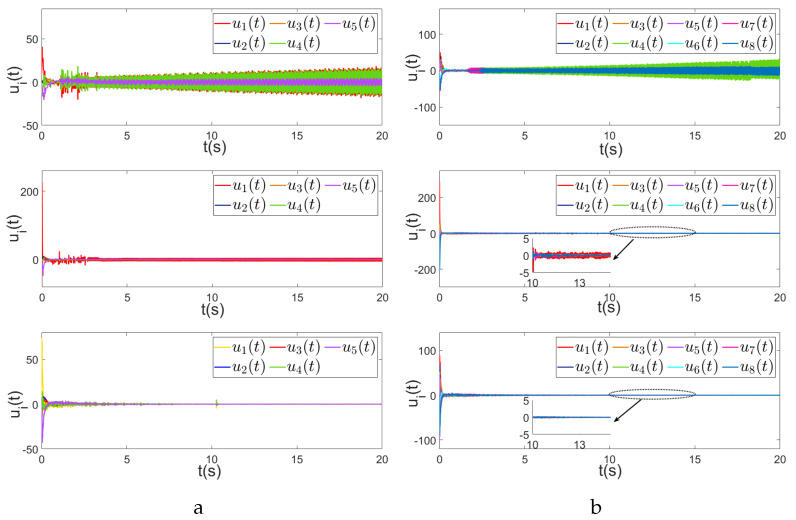
Control outputs. (**a**) Control outputs of the five agents, using the strategies in [31,35] and this paper. (**b**) Control outputs of the eight agents, using the strategies in [31,35] and this paper.

**Figure 6:**
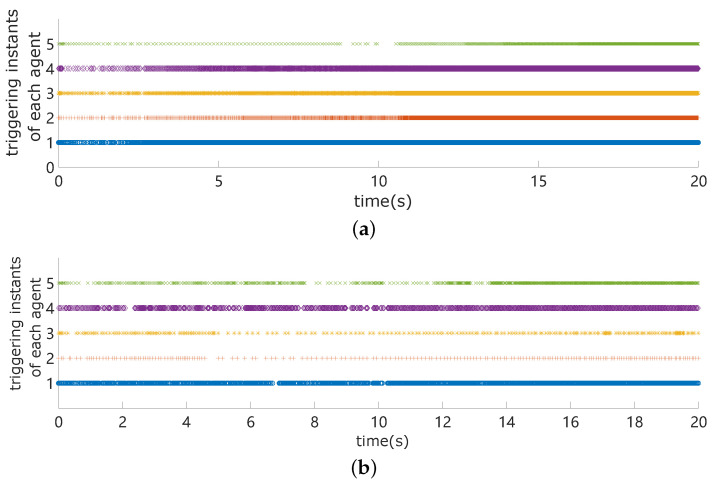
Triggering instants of agent *i*, i=1,…,5. (**a**) The triggering instants of agent *i* under the control strategy proposed in [35]; (**b**) The triggering instants of agent *i* under the control strategy proposed in this paper.

By using the three methods, we also compare the control outputs of the agents in Figure 5. We can see that both the control outputs using the proposed strategy and that in [35] have less fluctuation than [31]. However, our proposed method has fewer triggering times than [35], as shown in Figure 6.

Table 1 and Table 2, respectively, show the statistics of the triggering times of five and eight agents, using the three control strategies. According to Table 1 and Table 2, we can observe that the triggering frequency is significantly reduced by using our proposed controller, compared with that in papers [31,35].

In order to show the scalability of the proposed strategy, we consider two scenarios, i.e., one agent joins (leaves) the group of agents at a certain time instant. We reconsider the MAS represented by the topology in Figure 2.

**Figure 7:**
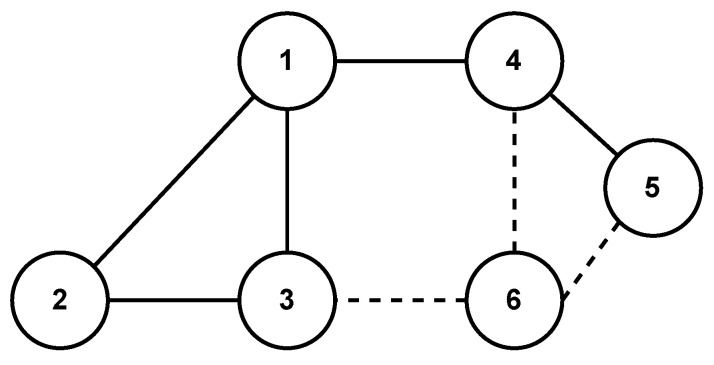
Undirected network topology of the MASs with five agents where 6th agent joints at 3 s.

**Figure 8:**
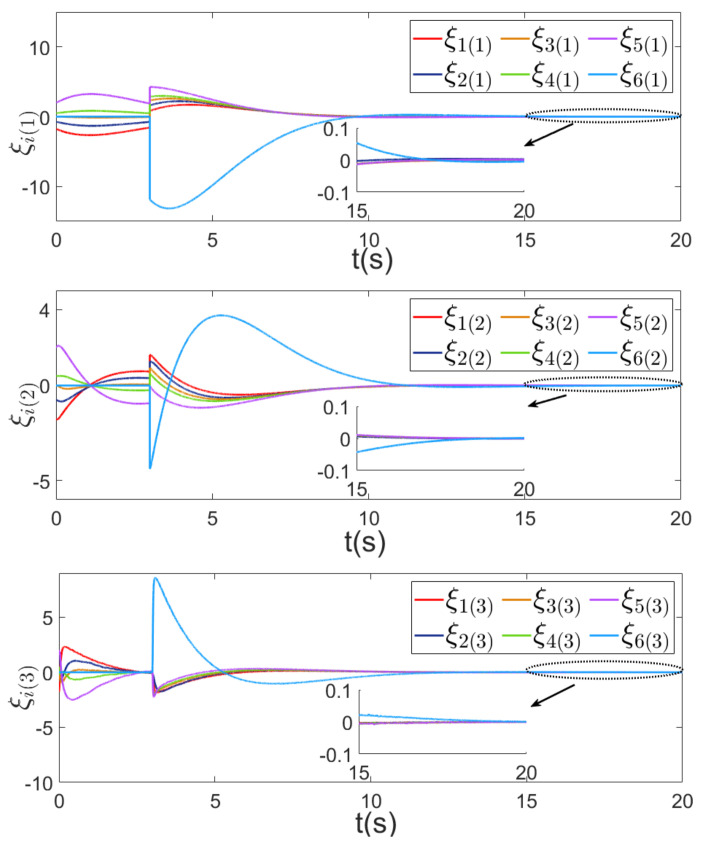
Consensus errors $\xi_{i},i=1,\ldots ,6$ under the control strategy of this paper.

**Figure 9:**
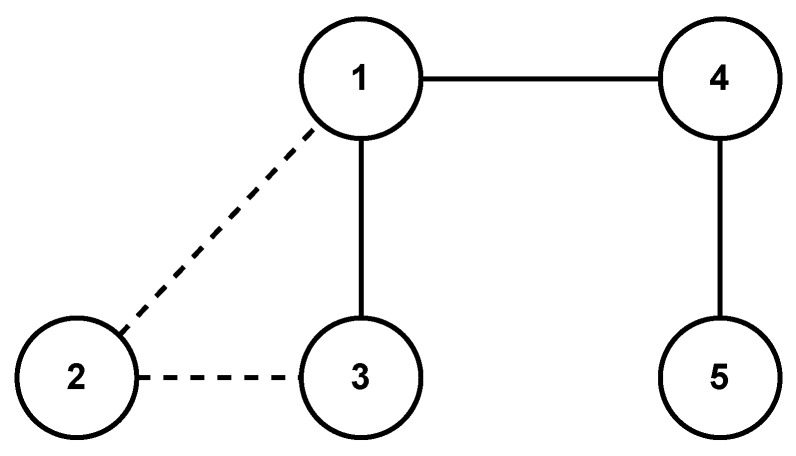
Undirected network topology of the MASs with five agents where 2nd agent is disconnected at 3 s.

**Figure 10:**
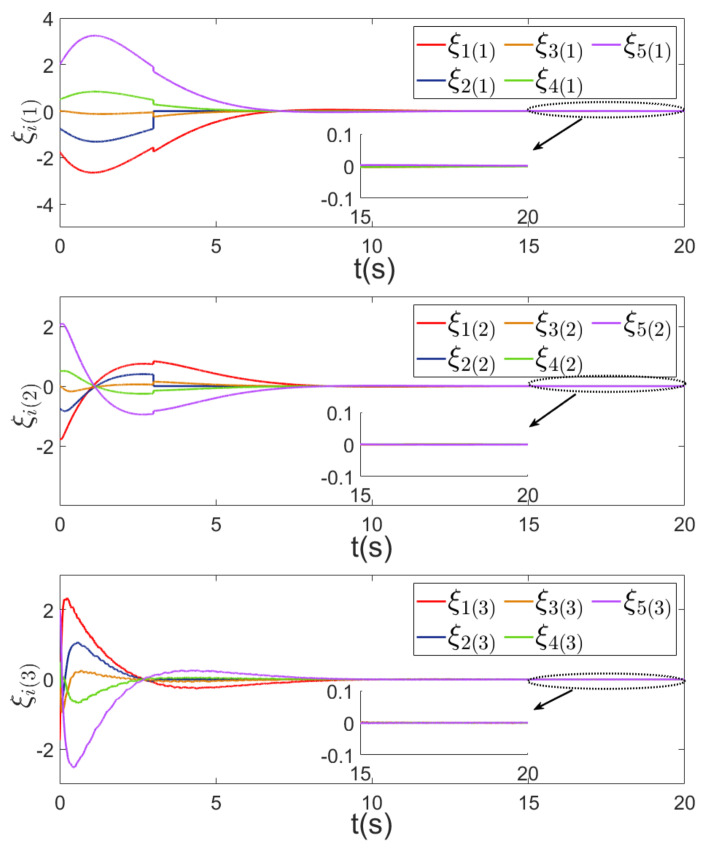
Consensus errors $\xi_{i},i=1,\cdots ,5$ under the control strategy of this paper.

In the first scenario, the 6th agent joints the group at 3 s. Then, the topology of the MAS becomes Figure 7. By using the proposed strategy, the consensus errors ξ are shown in Figure 8, in which we can observe that the MAS achieves consensus asymptotically even if the 6th agent joints to the network at 3 s. In the second scenario, the 2nd agent is disconnected with the other agents at 3 s. Then, the topology of the MAS becomes Figure 9. The consensus error ξ is shown in Figure 10, in which we can observe that the MAS also achieves consensus asymptotically by using the proposed strategy, even if the 2nd agent is disconnected with neighbors at 3 s.

## 5. Conclusions and Perspectives

In this paper, we address the consensus problem of multi-agent systems with general linear dynamics. A dynamic event-triggered adaptive control strategy is proposed. Compared with the existing works, our proposed strategy leads to a consensus of the agents’ states in the sense of asymptotic convergence. Furthermore, it improves the convergence speed and reduces the triggering frequency. The proposed strategy is fully distributed and scalable. Global information is not required by using the strategy. The agent states achieve consensus asymptotically even if the communication topology is switching. Under this strategy, continuous communication among agents and simultaneous broadcasts of the neighbors’ information are avoided.

In practice, time delay widely exists in discontinuous communications. The consensus problem with an uncertain and stochastic communication delay is an open topic for further study. On the other hand, the actuator saturation of agents should be considered, which leads to nonlinearities in the agent dynamics. In this case, the contradiction of triggering frequency and the response rapidity should be treated in the future.

## Figures and Tables

**Table 1:** The number of events driven by event triggers in MAS with five agents.

Agents	1	2	3	4	5	Total
Under [31]	2398	92	143	2398	173	5204
Under [35]	3746	2671	2721	2558	555	12,251
Proposed control	999	199	230	990	613	3031

**Table 2:** The number of events driven by event triggers in MAS with eight agents.

Agents	1	2	3	4	5	6	7	8	Total
Under [31]	1529	187	220	1526	237	129	139	1514	5481
Under [35]	2351	822	1420	681	453	1092	394	456	7669
Proposed control	544	462	404	644	481	308	450	516	3809

## Data Availability

Data are contained within the article.
